# *In Vitro*-Activity of Er:YAG Laser in Comparison with other Treatment Modalities on Biofilm Ablation from Implant and Tooth Surfaces

**DOI:** 10.1371/journal.pone.0171086

**Published:** 2017-01-26

**Authors:** Sigrun Eick, Ivan Meier, Florian Spoerlé, Philip Bender, Akira Aoki, Yuichi Izumi, Giovanni E. Salvi, Anton Sculean

**Affiliations:** 1 School of Dentistry, Department of Periodontology, University of Bern, Bern, Switzerland; 2 Graduate School of Medical and Dental Science, Tokyo Medical and Dental University, Tokyo, Japan; The Ohio State University, UNITED STATES

## Abstract

**Background and Aim:**

Bacterial biofilms play a major role in the etiology of periodontal and peri-implant diseases. The aim of the study was to evaluate the removal of bacterial biofilms and attachment of epithelial cells (EC), gingival fibroblasts (GF) and osteoblast-like cells (OC) to dentin and titanium surfaces after Er:YAG laser (Er:YAG) in comparison with other treatment methods.

**Material and Methods:**

Multi-species bacterial biofilms were grown on standardized dentin and titanium specimens with a sand-blasted and acid etched (SLA) surface for 3.5 d. Thereafter, the specimens were placed into artificially-created pockets. The following methods for biofilm removal were used: 1) Gracey (dentin) or titanium curettes (CUR), 2) Er:YAG, 3) photodynamic therapy (PDT) and 4) CUR with adjunctive PDT (CUR/PDT). Colony forming units (CFUs) of the remaining biofilms and attachment of EC, GF and OC were determined. Statistical analysis was performed by means of ANOVA with post-hoc LSD.

**Results:**

All treatment methods decreased statistically significantly (p<0.001) total CFUs in biofilms compared with untreated dentin and titanium surfaces respectively. On dentin, Er:YAG was equally efficient as CUR and PDT but inferior to CUR/PDT (p = 0.005). On titanium, surfaces, the use of Er:YAG resulted in statistically significantly superior biofilm removal compared to the 3 other treatments (each p<0.001). Counts of attached EC, GF and OC were the lowest on untreated contaminated dentin and titanium surfaces each. After CUR/PDT higher EC counts were found on dentin (p = 0.006). On titanium, all decontamination methods statistically significantly increased (p<0.001) the counts of attached EC without differences between groups. Statistically significantly higher counts of GF (p = 0.024) and OC (p<0.001) were observed after Er:YAG decontamination compared with untreated surfaces.

**Conclusion:**

Ablation of subgingival biofilms and in particular decontamination of titanium implant surfaces with an Er:YAG laser seem to be a promising approach and warrants further investigations.

## Introduction

Lasers have been introduced in dentistry in the 60ies of last century. In one of the first reports a ruby laser with an energy density of about 9’000 J / cm^2^ was applied for destruction of carious lesions in in-vitro experiments [1]. Meanwhile, lasers were introduced in nearly all fields of dentistry including endodontics, periodontology, implantology, oral surgery, orthodontics and cariology [2]. The term “LASER” stands for “Light Amplification by Stimulated Emission of Radiation”. The principle is that electrons being normally in a low energy state are transferred to a high energy state; when moving back to the low energy state the absorbed energy is released. Lasers used in dentistry are differentiated between low-intensity (*e*.*g*. diode lasers) and high-intensity lasers (*e*.*g*. Erbium-Doped: Yttrium, Aluminum, Garnet (Er:YAG) laser). In periodontal therapy low-intensity lasers are discussed to improve wound healing and combined with photosensitizer (photodynamic therapy) they can act exert antimicrobial activity [3].

Er:YAG lasers with a wavelength of 2’940 nm can be used both for soft and hard-tissue surgery as well as for removal of subgingival calculus from periodontal pockets. Its laser light is absorbed in superficial layers and thus Er:YAG laser does not penetrate deeply into the tissues. The efficacy in soft and hard tissue is explained as evaporation by photo-thermal interactions [4]. Er:YAG laser was proven to act antimicrobial. When applying to microbes spread on agar plates a clear inhibition zone was found both for microbes being associated with endodontic (e.g. *Candida albicans*, *Enterococcus faecalis*) [5] and periodontal infections (e.g. *Porphyromonas gingivalis*) [6].

Treatment of periodontal and peri-implant diseases includes various anti-infectious regimens. The primary goal is to remove hard and soft bacterial deposits leading to a smooth and biocompatible surface in order to minimize further bacterial adhesion and to facilitate host cell re-attachment. In the treatment of periodontal disease, scaling and root planing (SRP), which includes mechanical removal of biofilms, is an effective causative method for infection control [7]. On the other hand, in peri-implant diseases, mechanical non-surgical therapy alone may be effective in the management mucositis but not of peri-implantitis lesions [8].

Er:YAG lasers have been often discussed as a treatment options for removal of subgingival and peri-implant biofilms; available evidence suggests that subgingival and submucosal debridement with Er:YAG laser treatment may reduce periodontal and peri-implant mucosal inflammation [9,10,11]. In periodontitis treatment outcomes, a recent systematic review indicated that Er:YAG laser was as effective as scaling and root planing 3 months after therapy [12]. In peri-implantitis treatment, a meta-analysis including data of four randomized clinical trials found short-term benefits in terms of probing depth reduction after Er:YAG but a general superiority to mechanical debridement was not observed [13].

Subgingival and peri-implant biofilms are communities consisting of numerous bacteria. Organisms strongly being associated with periodontal disease include *Aggregatibacter actinomycetemcomitans*, *Porphyromonas gingivalis*, *Tannerella forsythia* and *Treponema denticola* [14,15]. Bacteria associated with peri-implant lesions are at least partially the same as in periodontitis. Black pigmented *Prevotella* species, *A*. *actinomycetemcomitans*, *P*. *gingivalis* were assessed in higher quantities from peri-implantitis lesions than from healthy controls [16,17]. In patients having a history of treated gingivitis or periodontitis, periodontopathogens (*P*. *gingivalis*, *T*. *forsythia*, *T*. *denticola*, *Fusobacterium nucleatum*, *Prevotella intermedia*) were already detectible 30 minutes after implant placement [18] and one week following abutment connection [19]. In a retrospective cross-sectional study analyzing implants and the adjacent teeth 10 years after placement, more *T*. *forsythia*, *Parvimonas micra*, *F*. *nucleatum / necrophorum* and *Campylobacter rectus* were found at implants than at teeth [20]. The counts correlated positively for all these species at implants and teeth and a high correlation was observed for *P*. *gingivalis* (R = 0.503) [20]. Taken together, the available data from the literature clearly suggest, that at present, there is a stringent need for developing novel approaches for predictable decontamination of implant surfaces in order to determine the best suitable method for biofilm removal and creation of a surface favoring adhesion of epithelial cells, gingival fibroblasts, and osteoblast-like cells.

Therefore, the aims of the present in-vitro study were: (i) to evaluate the activity of Er:YAG laser in killing selected planktonic microorganisms and to compare its efficacy with that of other treatment modalities (hand instrumentation with using curettes, photodynamic therapy, hand instrumentation combined with photodynamic therapy) on the destruction and removal of a 12-species biofilm on dentin and titanium surfaces and (ii) to evaluate and compare the adhesion of epithelial cells, gingival fibroblasts, and osteoblast-like cells on the dentin and titanium surfaces following destruction and removal of a 12-species biofilm following Er:YAG laser application and other treatment modalities.

## Materials and Methods

### Microorganisms

The following bacterial strains were included in the study: *Streptococcus gordonii* ATCC 10558, *Actinomyces naeslundii* ATCC 12104, *F*. *nucleatum* ATCC 25586, *Campylobacter rectus* ATCC 33238, *Filifactor alocis* ATCC 35896, *Eikenella corrodens* ATCC 23834, *P*. *intermedia* ATCC 25611, *P*. *micra* ATCC 33270, *P*. *gingivalis* ATCC 33277, *T*. *forsythia* ATCC 43037, *T*. *denticola* ATCC 35405 and *A*. *actinomycetemcomitans* Y4.

Before an experiment, all strains (except for *T*. *denticola* ATCC 35405) were precultivated on Schaedler agar plates (Oxoid, Basingstoke, UK) with 5% sheep blood in an anaerobic atmosphere or with 5% CO_2_ (*A*. *actinomycetemcomitans* Y4 and *S*. *gordonii* ATCC 10558). *T*. *denticola* was maintained in modified mycoplasma broth (BD, Franklin Lake, NJ) added by 1 mg/ml glucose, 400 μg/ml niacinamide, 150 μg/ml spermine tetrahydrochloride, 20 μg/ml Na isobutyrate enriched with 1 g/ml cysteine and 5 μg/ml cocarboxylase in anaerobic conditions.

### Antimicrobial activity on planktonic bacteria

A defined inoculum of microorganisms (10^6^ and 10^3^ in 100 μl RPMI 1640 without phenol red) was prepared and pipetted on glass slides which were transferred to a 50 ml tube. Thereafter, Er:YAG laser (Erwin AdvErl, J. Morita) was applied with irradiation powers 30 mJ, 50 mJ and 70 mJ (each on panel)-20 pps for 10 s, 20 s and 3 x 20 s (equivalent to 0.6 W, 1 W, 1.4 W). The numbers of colony forming units (cfu) were determined after addition of NaCl 0.9% solution, preparing a 10-fold dilution series and plating of each 100 μl on agar-plates. In these experiments selected single species were tested in independent replicates.

### Test specimens for biofilm formation

Dentin specimens were prepared from porcine teeth being a by-pass product in slaughterhouse. Teeth were removed from the jaws and placed in chloramine solution for disinfection. The crowns of the teeth were removed and dentin slices of the buccal side of the roots were cut with diamond disks (~ 6 × 12 mm) with a thickness of ~3 mm. The surface properties of the buccal side of the dentin specimens were standardized by grinding with silicon carbide papers of #2400 grit size, corresponding to an abrasive particle size of 6.5 μm. Finally the dentin slices were fixed on plastic disks as described recently [21].

Titanium specimes with a sandblasted and acid-etched (SLA) surface (Institut Straumann AG, Basel, Switzerland) were fixed on plastic specimens similarly as described above.

### Biofilm formation

The dentin and titanium specimens were covered with a 5% chicken serum albumin solution for 1h and thereafter placed in tubes. Suspensions of 12 bacterial strains were mixed with nutrient broth and transferred to the tubes. After incubation in anaerobic conditions for 72 h, two thirds of the medium were exchanged and *P*. *gingivalis* ATCC 33277, *T*. *forsythia* ATCC 43037 and *T*. *denticola* ATCC 35406 were be added again. After final incubation of 48 h, the medium was removed and the treatment methods were applied.

### Methods for biofilm removal

Plastic specimens with the dentin or titanium disks were transferred in the pocket model as described previously [21]. In this model, instrumentation was performed obliquely to the dentin and titanium surfaces thus mimicking as close as possible the clinical situation. Standardized test specimens were used allowing a comparison between the different treatment methods and in addition between different surfaces like dentine and titanium. However, it should be noted, that the curved shape of teeth, a potential coating with calculus as well as the niches and the different surfaces of a dental implant were not considered.

In biofilm experiments irradiation power was 50 mJ (on panel)-20 Hz per pulse for titanium and 70 mJ (on panel)-20 Hz per pulse for dentin. The energy was chosen based on the manufacturer’s recommendation; higher energy was shown to damage the titanium surface [22]

For all Er:YAG laser applications, the PS600 T tip was used in the respective handpiece. The laser was used in an oblique angle for 15 s with concomitant water spray irrigation (5 ml / min) in contact with the surface of the dentine and in non-contact with the surface of titanium disk.

Three positive control treatments were used. In case of dentin specimens, Gracey curettes (CUR) made of stainless steel (Deppeler SA, Rolle, Switzerland) while in case of titanium plates, the instrumentation was performed by means of titanium curettes (Deppeler SA, Rolle, Switzerland). Specimens were instrumented from apically to coronally by means of 20 strokes using both sites of the curettes. Each side of the curette was used for five specimens and then replaced by a new curette. Before instrumentation, after every 10th stroke and at the end of instrumentation, the dentin and titanium surfaces in the pockets were rinsed with 2.5 ml of 0.9% w/v NaCl. The second positive control was photodynamic therapy (PDT) applying 25 μl of phenothiazine chloride (HELBO^®^ Blue Photosensitizer; Photodynamic Systems GmBH, Wels, Austria) as photosensitizer for 3 min combined with 2 × 10 s of a hand-held diode laser (HELBO^®^ 3D Pocket, Photodynamic Systems GmbH) with a wavelength of 660 nm and a power density of 100 mW according to the manufacturer’s instruction before final rinsing with 2.5 ml of 0.9% w/v NaCl. The third positive control combined CUR (10 strokes) and PDT as described above (CUR/PDT).

Untreated contaminated specimens served as negative controls (con). Additionally, in cell adhesion assays, non-contaminated specimens were included as controls (con cells).

### Influence of treatment methods on bacteria

Immediately after treatments, biofilm samples were collected from the surface and suspended in 0.9% w/v NaCl solution. After making a serial dilution each 25 μl was spread on Schaedler agar plates, incubated in anaerobic conditions and the total colony forming units (cfu) counts incl. black-pigmented were assessed. Further, the loads of *P*. *gingivalis*, *T*. *forsythia*, *P*. *micra* were determined by using real-time PCR [23] and those of *F*. *nucleatum* by counting relevant CFU.

### Influence of biofilm removal on adhesion of epithelial cells, gingival fibroblasts and osteoblast-like cells

Epithelial TIGK cells donated by R Lamont, University of Louisville, USA were used. Cells were maintained in Keratinocyte growth medium (Ruwag Handels AG, Bettlach, Switzerland). Human alveolar osteoblasts (HAO) as well as human gingival fibroblasts were obtained from periodontally healthy patients during surgery (extraction of teeth for orthodontic reasons) and processed as described recently [24]. Cells were anonymously collected from patients during regular periodontal or implant treatment following written informed consent. This procedure is approved by the Ethics Committee of the University of Bern. The cultivation medium was DMEM (DMEM, Invitrogen, Carlsbad, CA) 0% of fetal bovine serum (FBS, Invitrogen). For experiments, both cell types were used from passages 4–6.

After applying the different treatment methods and removing the test specimens from the artificial pockets, the dentin and titanium disks were carefully removed from the plastic specimens. Before seeded into cell-culture-well-plates with the respective cells disks were exposed to UV to avoid bacterial overgrowth in the cell culture media [21]. About 5 × 10^4^ cells per well were added. Cells were incubated with 5% CO_2_ for 72 h. Afterwards, cells were fixed and stained with DAPI (Roche Diagnostics GmbH, Mannheim, Germany) for cells being exposed to titanium and with Pappenheim (Hemacolor^®^, Merck Millipore, Darmstadt, Germany) for those being exposed to dentin. Ten fields of 1 mm^2^ each were counted. Fields were equally distributed from the whole slide.

All biofilm experiments were conducted in independent quadruplicates in two series.

### Statistical analysis

Data used in statistical analysis are presented as mean and standard deviation (SD). In case of bacterial counts log_10_ values are reported. Data were compared using a one-way analysis of variance (ANOVA) with post-hoc comparisons of groups using LSD corrections. A *p*-value of 0.05 was considered to be statistically significant. SPSS software (version 22.0) was used for statistical analysis.

## Results

### Planktonic bacteria

**Fig 1:**
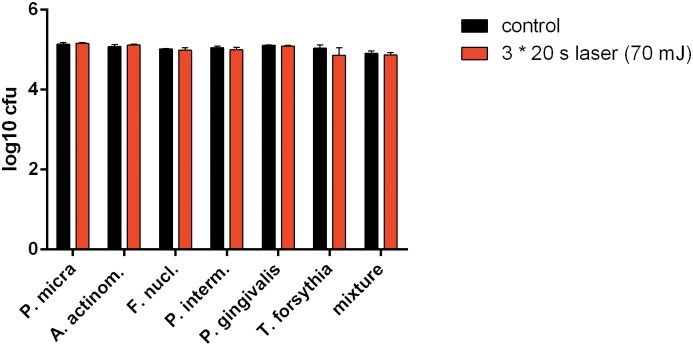
Killing of planktonic bacteria. Killing of selected bacterial species and a 12-species mixture after 3*20 s of laser irradiation with a power of 70 mJ.

As no dependence of the irradiation power was visible in first experiments, only the highest power (70 mJ) and longest time (3 times 20 s) were used in the follow-up experiments. However, differences to untreated control did not exceed 0.15 log_10_ CFU (Fig 1).

### Biofilm removal from dentin disks

**Fig 2:**
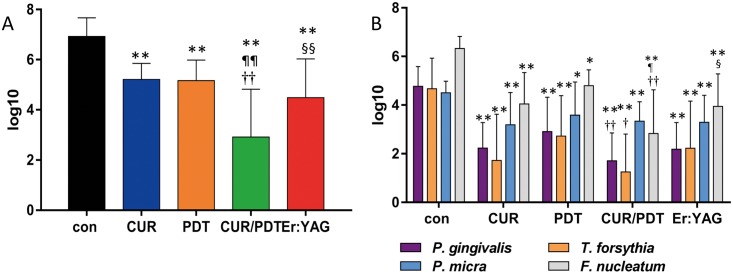
A-B. Biofilm removal from dentine disks. Remains of biofilm after exposing to Gracey curette (CUR), photodynamic therapy (PDT), CUR combined with PDT (CUR/PDT) and Er:YAG laser irradiation (Er:YAG) Presented are total counts (cfu; A) and counts for selected species (B) * p<0.05; ** p<0.01 compared with control (con) ^¶^ p<0.05; ^¶¶^ p<0.01 compared with CUR ^†^ p<0.05; ^††^ p<0.01 compared with PDT ^§^ p<0.05; ^§§^ p<0.01 compared with CUR/PDT.

All treatment procedures reduced statistically significantly (each p<0.01) the total bacterial counts. Er:YAG reduced the bacterial counts by 2.44 log_10_ with no significant difference to CUR (reduction 1.71 log_10_). The lowest values of remained bacteria (reduction by 4.01 log_10_) were seen for CUR/PDT each being significantly different (p<0.01) to all other treatments incl. Er:YAG (p = 0.005). The analyzed single species were also statistically significantly reduced in comparison with the untreated control. Counts of *P*. *gingivalis* (p = 0.008), *T*. *forsythia* (p = 0.026) and *F*. *nucleatum (*p = 0.002) were statistically significantly lower after CUR/PDT than after applying PDT alone (Fig 2).

### Adhesion of gingival epithelial cells and PDL fibroblasts after biofilm removal to dentin disks

Attachment of gingival epithelial cells dropped down close to zero if the biofilm was not removed (difference to con cells p<0.001). After treatment of the surface, the numbers increased but the difference to the untreated biofilm-exposed sample was only statistically significant in the treatment performed with CUR/PDT (p = 0.006). Except for CUR/PDT, following all other treatments, the significance to con cells remained (each p<0.05).

**Fig 3:**
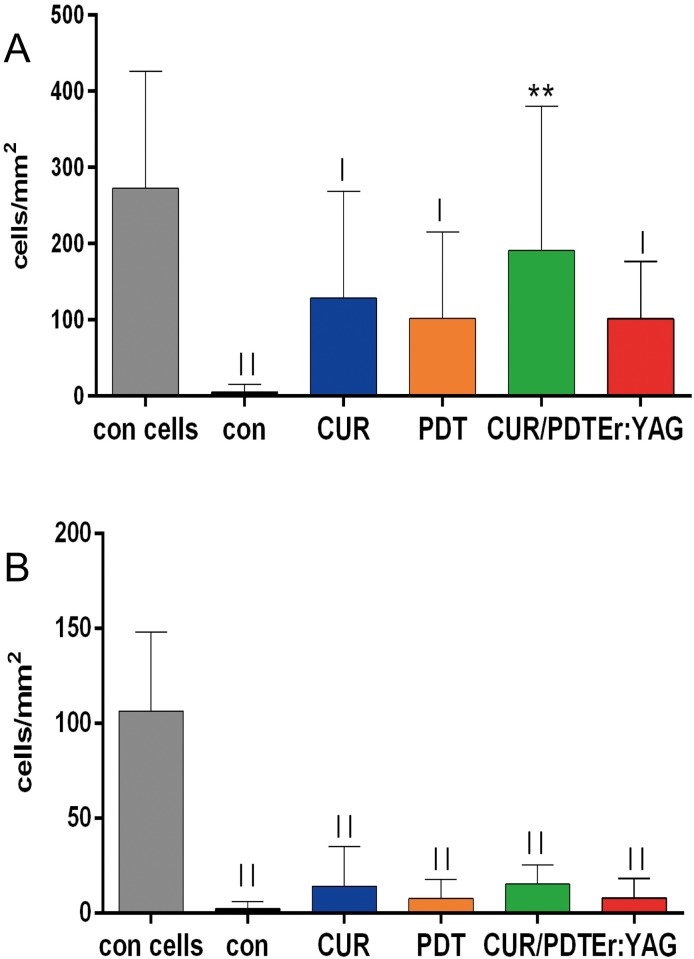
A-B. Adhesion of cells after biofilm removal from dentine disks. Adhesion of gingival epithelial cells (A) and PDL fibroblasts (B) after biofilm removal by applying Gracey curette (CUR), photodynamic therapy (PDT), CUR combined with PDT (CUR/PDT) and Er:YAG laser irradiation (Er:YAG) ** p<0.01 compared with control with bacteria (con) ^|^ p<0.05; ^||^ p<0.01 compared with control without bacteria (con cells).

The number of PDL fibroblasts dropped also down after exposing them to biofilms (p<0.001). No clear increase in their counts was seen after any treatment and the difference to con cells remained (each p<0.001) (Fig 3).

#### Biofilm removal on titanium disks

**Fig 4:**
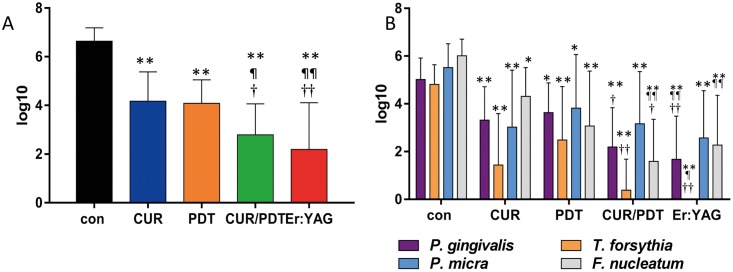
A-B. Biofilm removal from titanium disks. Remains of biofilm after applying titanium curette (CUR), photodynamic therapy (PDT), titanium curette combined with PDT (CUR/PDT) and Er:YAG laser irradiation (Er:YAG) Presented are total counts (cfu; A) and counts for selected species (B) * p<0.05; ** p<0.01 compared to control (con) ^¶^ p<0.05; ^¶¶^ p<0.01 compared to CUR ^†^ p<0.05; ^††^ p<0.01 compared to PDT.

All treatment procedures reduced statistically significantly (each p<0.01) the total bacterial counts. The lowest values of remaining bacteria (reduction by 4.45 log_10_) were seen for Er:YAG laser each being statistically significantly different (p<0.01) to PDT and CUR (total counts). Moreover, counts of *P*. *gingivalis* and *T*. *forsythia* were less numerous than following PDT alone and CUR (Fig 4).

#### Adhesion of gingival epithelial cells, gingival fibroblasts and osteoblast-like cells after biofilm removal to titanium disks

Attachment of gingival epithelial cells dropped down if the biofilm was not removed (difference to con cells p<0.001). Following any of the used treatments, the numbers increased statistically significantly (p<0.01 except for CUR/PDT (p = 0.016)). A statistical significant difference to cell adherence to titanium surfaces without bacterial biofilm exposure was observed (each p<0.001).

The number of PDL fibroblasts decreased after exposing them to biofilms (p = 0.048). The difference was also statistically significant when PDT was applied for biofilm removal (p = 0.016). A clear increase in their counts was seen following treatment with Er:YAG and the difference was statistically significant compared to con (p = 0.024) and PDT (p = 0.007).

**Fig 5:**
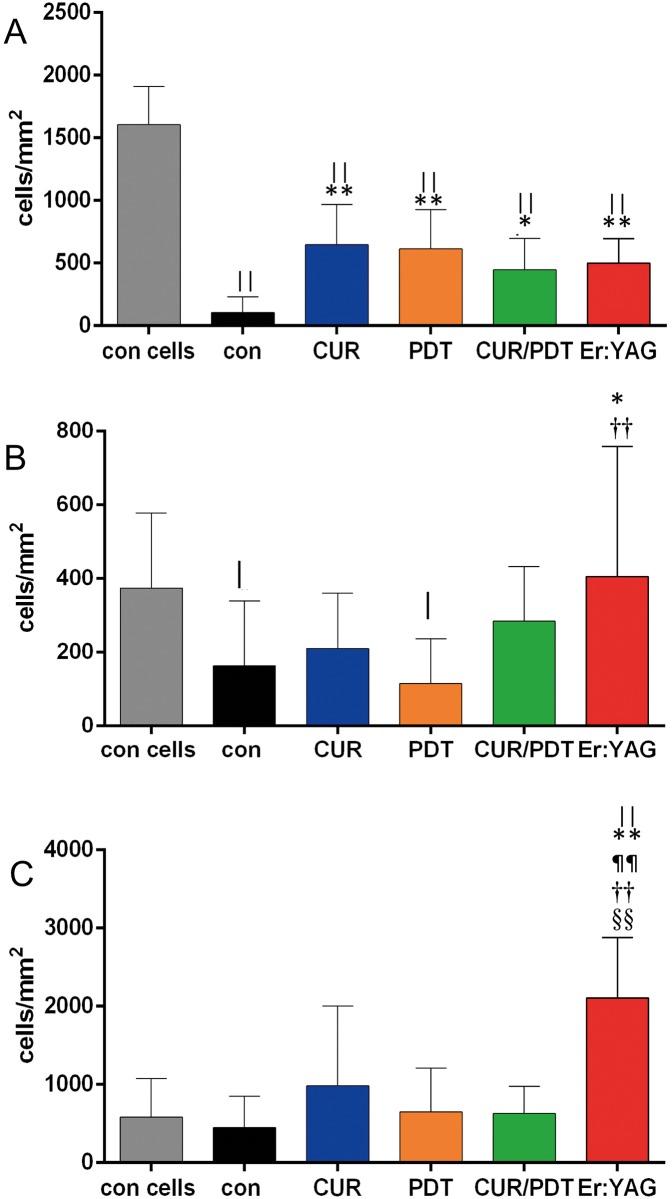
A-C. Adhesion of cells after biofilm removal from titanium disks. Adhesion of gingival epithelial cells (A), gingival fibroblasts (B) and osteoblast-like cells (C) before and after biofilm removal by after applying titanium curette (CUR), photodynamic therapy (PDT), titanium curette combined with PDT (CUR/PDT) and Er:YAG laser irradiation (Er:YAG) * p<0.05; ** p<0.01 compared with control with bacteria (con) ^|^ p<0.05; ^||^ p<0.01 compared with control without bacteria (con cells) ^¶¶^ p<0.01 compared with CUR ^††^ p<0.01 compared with PDT ^§§^ p<0.01 compared with CUR/PDT.

The number of the attached osteoblasts did not appear to be affected by exposing titanium to bacterial biofilm. However, following removal of biofilm with Er:YAG laser, the osteoblasts adhered in a statistically significant higher number (each p<0.01) compared with any of the controls or other treatment groups (Fig 5).

## Discussion

The present *in-vitro* study has evaluated the application of Er:YAG laser in comparison with hand instruments and photodynamic therapy in ablation of periodontal and peri-implant biofilms.

The first series of experiments focused on the activity of Er:YAG laser against planktonic bacteria. Up to now, the bactericidal activity of Er:YAG laser on oral bacteria was only very rarely investigated. The missing bactericidal activity on planktonic bacteria is in contrast with a few previous studies reporting bactericidal activity [6,25,26]. In our study, the bacteria were placed on glass slides before laser irradiation. Since Er:YAG laser can only be used in conjunction with water cooling (i.e. irrigation), it cannot be completely ruled out that, at least some bacteria were swept off fast and thus were not directly exposed to the laser beam. However, when Er:YAG laser was used without water cooling (data not shown) no bactericidal activity was observed. In some other studies, Er:YAG laser was applied to bacteria on agar plates [6,25], and therefore an absorption of the laser by the agar and evaporation of the agar might have also influenced the results. In one study where microorganisms were spread on agar or were incorporated in the agar, only superficially located microorganisms and not those located in deeper layers, were affected by Er:YAG laser [5]. This finding suggests that the reduction of bacterial counts in biofilms is rather ablative than bactericidal.

In this study biofilms of comparable composition were established both on dentin and titanium disks, thus allowing direct comparison of the used methods. Well defined multi-species subgingival/periodontal biofilms on dentin and titanium disks were exposed to Er:YAG laser irradiation and compared with other treatment modalities including hand instrumentation alone. The reason to create an artificial “pocket” was to reproduce the clinical situation of narrow subgingival areas, thus enabling to perform the treatments in a way as close to the clinical situation as possible [21].

The only difference between the untreated biofilm on dentin and titanium surfaces was in the counts of *P*. *micra*, the species was found in higher numbers on titanium than on dentin disks (difference 1.07 log_10_; p = 0.005). In contrast to the missing bactericidal activity, ablation of biofilms from both dentin and titanium specimens by means Er:YAG laser was clearly observed. The differences to the untreated controls were 2.44 log_10_ for dentin and 4.45 log_10_ for titanium surfaces, respectively. On dentin, Er:YAG was as efficient as hand instrumentation alone. Reduction of viable counts within single-species biofilms (*P*. *gingivalis*, *F*. *nucleatum*, *A*. *naeslundii* and others) on hydroxyapatite disks by this laser type irradiating with 20–80 mJ / puls at 10 pps for 10 s has been reported previously [26] but superiority to other methods was not observed. This is in contrast to another study where extracted teeth were exposed to ultrasonication and Er:YAG laser [6]. In that study [6] at teeth partly covered with calculus, the total anaerobic counts in biofilm controls were only 3.71 log_10_ while comparison was made to ultrasonication. In the present study, ultrasonication was not included but in a recent study with similar methodology superiority of ultrasonication to hand instrumentation was reported [21].

Whereas no statistically significant differences were found compared to hand instrumentation on dentin, Er:YAG was most effective in reducing bacterial counts in biofilms on titanium. The difference between the two types of test specimens (dentin and titanium) was 2.02 log_10_ (p = 0.03). Er:YAG laser removed more efficiently early plaque than plastic curettes combined with chlorhexidine rinsing and ultrasonication from titanium disks embedded in acrylic splints worn by volunteers [27]. In vitro, Er:YAG decreased significantly viability of *Candida albicans* biofilm by using 100 mJ and an irradiation time of 80 s [28]. The high biofilm ablation by Er:YAG laser might be of particular importance, as in vivo the niches formed by the screw of the abutment cannot be reached by hand instruments.

Among the other tested methods, combination of CUR/PDT with a reduction of about 4 log_10_ was always more efficient than CUR or PDT alone, on both dentin and titanium. Several studies reported an additional improvement of clinical parameters when photodynamic therapy was applied additionally to scaling and root planing in the treatment of periodontitis [29,30]. Moreover, in the treatment of initial peri-implantitis, adjunctive photodynamic therapy was as effective as local antibiotic delivery in the reduction of sites with bleeding on probing [31].

Adhesion of host cells correlated inversely with the remained bacterial counts on the surface after various treatments. The lowest counts of epithelial cells were seen when non-disrupted biofilm was present, the highest without any previous bacterial contamination. Despite the fact that bacteria were inactivated by UV-light which is used to stop multiplication, virulence factors might still have been present. For example, *P*. *gingivalis* proteases can degrade a number of cell adhesion molecules and thus induce detachment and apoptosis of epithelial cells (Chen, Casiano *et al*. 2001). While any bacterial constituent clearly inhibited attachment of PDL fibroblasts to dentin, after treatment of titanium surfaces with Er:YAG laser, no differences in the attachment of gingival fibroblasts were observed compared to pristine test surfaces (i.e. a surfaces that have not been exposed to any bacterium). This is confirmatory to another in-vitro study where decontamination of a single-species biofilm of *P*. *gingivalis* by Er:YAG laser at pulse energy 60 mJ and frequency 10 pps in vitro did not interfere with growth of gingival fibroblasts thereafter [32]. Moreover, in beagle dogs the Er:YAG laser was applied to implants placed into the mandible without disturbing osseointegration [22].

An important finding of the present study was the high adhesion rate of osteoblast-like cells on the Er:YAG treated titanium surfaces which was even higher than that observed on pristine test specimens without any bacteria. This finding confirms previous reports on the good adhesion of a osteoblast-like cell line [27] and primary osteoblast-like cells [33] after decontamination of SLA titanium surface using Er:YAG laser.

Er:YAG laser has the highest absorption in water and a very high absorption in hydroxyapatite [34]. Therefore, it may be anticipated that the absorption of this laser on titanium is much lower and following, more energy can be absorbed by a biofilm that contains a high percentage of water. This phenomenon might in turn lead to biofilm ablation. Temperature in the closed surrounding of the titanium implant increase after 60 s Er:YAG irradiation 100 mJ 20 Hz by 10.5°C with air/water [35]. Below 0.5 mm dentin rise in temperature is 3.86°C when irradiating with 12.7 mJ / cm^2^ 20 Hz for 30 s with water cooling [36]. Micro-rough SLA surfaces at titanium are not damaged up to 140 mJ per pulse [37]. After Er:YAG irradiation with 100 mJ 10 Hz for 1 min to SLA surfaces, the surface roughness decreased while wettability increased and as a consequence, the subsequently adhered osteoblast-like cells exhibited a higher cell-viability [38]. On dentin Er:YAG laser increased wettability but also surface porosities [39].

Most studies which have compared microbiologically and clinically the outcomes following scaling and root planing with Er:YAG laser debridement in patients with chronic periodontitis failed to reveal any statistically significant differences in any of the analyzed parameters [40,41]. In only one study a greater reduction of *P*. *gingivalis* was observed at 12 months following the use of Er:YAG laser [42]. Nonsurgical treatment of peri-implantitis lesions at implants with a machined surface by means of Er:YAG laser revealed minor clinical and microbiological changes, [43] while in a pilot study, Er:YAG laser showed greater reduction in bleeding on probing when compared with the use of plastic curettes and chlorhexidine rinsing [44]. On the other hand, when Er:YAG laser was used during surgery no superior improvements compared with those obtained following the use of plastic curettes were found [45].

## Conclusion

Within their limits, the present data indicate that: a) on dentin surfaces, Er:YAG laser appears to be equally effective as hand instrumentation for removing bacterial biofilms, b) the combination of CUR and PDT appears to be a suitable method to additionally decontaminate dentin surfaces, and c) on titanium surfaces, the use of Er:YAG laser yielded clear advantages compared to the other debridement modalities.

## Supporting Information

S1 File Supporting data to the Figs 1–5. The means and SD for Figs 1**–**5 incl. exact p-values are presented.
(DOCX)

## References

[1] Goldman L, Hornby P, Meyer R, Goldman B (1964) Impact of the Laser on Dental Caries. Nature 203: 417. 10.1038/203417a0 14197393

[2] Green J, Weiss A, Stern A (2011) Lasers and radiofrequency devices in dentistry. Dent Clin North Am 55: 585–597, ix–x. 10.1016/j.cden.2011.02.017 21726692

[3] Passanezi E, Damante CA, de Rezende ML, Greghi SL (2015) Lasers in periodontal therapy. Periodontol 2000 67: 268–291. 10.1111/prd.12067 25494605

[4] Aoki A, Mizutani K, Schwarz F, Sculean A, Yukna RA, Takasaki AA, et al (2015) Periodontal and peri-implant wound healing following laser therapy. Periodontol 2000 68: 217–269. 10.1111/prd.12080 25867988

[5] Meire MA, Coenye T, Nelis HJ, De Moor RJ (2012) In vitro inactivation of endodontic pathogens with Nd:YAG and Er:YAG lasers. Lasers Med Sci 27: 695–701. 10.1007/s10103-011-0940-z 21691826

[6] Akiyama F, Aoki A, Miura-Uchiyama M, Sasaki KM, Ichinose S, Umeda M, et al (2011) In vitro studies of the ablation mechanism of periodontopathic bacteria and decontamination effect on periodontally diseased root surfaces by erbium:yttrium-aluminum-garnet laser. Lasers Med Sci 26: 193–204. 10.1007/s10103-010-0763-3 20309597

[7] Apatzidou DA, Kinane DF (2010) Nonsurgical mechanical treatment strategies for periodontal disease. Dent Clin North Am 54: 1–12. 10.1016/j.cden.2009.08.006 20103469

[8] Renvert S, Roos-Jansaker AM, Claffey N (2008) Non-surgical treatment of peri-implant mucositis and peri-implantitis: a literature review. J Clin Periodontol 35: 305–315. 10.1111/j.1600-051X.2008.01276.x 18724858

[9] Muthukuru M, Zainvi A, Esplugues EO, Flemmig TF (2012) Non-surgical therapy for the management of peri-implantitis: a systematic review. Clin Oral Implants Res 23 Suppl 6: 77–83. 23062131 10.1111/j.1600-0501.2012.02542.x

[10] Tomasi C, Schander K, Dahlen G, Wennstrom JL (2006) Short-term clinical and microbiologic effects of pocket debridement with an Er:YAG laser during periodontal maintenance. J Periodontol 77: 111–118. 10.1902/jop.2006.77.1.111 16579711

[11] Yilmaz S, Kut B, Gursoy H, Eren-Kuru B, Noyan U, Kadir T (2012) Er:YAG laser versus systemic metronidazole as an adjunct to nonsurgical periodontal therapy: a clinical and microbiological study. Photomed Laser Surg 30: 325–330. 10.1089/pho.2010.2762 22509738

[12] Zhao Y, Yin Y, Tao L, Nie P, Tang Y, Zhu M (2014) Er:YAG laser versus scaling and root planing as alternative or adjuvant for chronic periodontitis treatment: a systematic review. J Clin Periodontol 41: 1069–1079. 10.1111/jcpe.12304 25164559

[13] Yan M, Liu M, Wang M, Yin F, Xia H (2015) The effects of Er:YAG on the treatment of peri-implantitis: a meta-analysis of randomized controlled trials. Lasers Med Sci 30: 1843–1853. 10.1007/s10103-014-1692-3 25428598

[14] Henderson B, Nair SP, Ward JM, Wilson M (2003) Molecular pathogenicity of the oral opportunistic pathogen Actinobacillus actinomycetemcomitans. Annu Rev Microbiol 57: 29–55. 10.1146/annurev.micro.57.030502.090908 14527274

[15] Holt SC, Ebersole JL (2005) Porphyromonas gingivalis, Treponema denticola, and Tannerella forsythia: the "red complex", a prototype polybacterial pathogenic consortium in periodontitis. Periodontol 2000 38: 72–122. 10.1111/j.1600-0757.2005.00113.x 15853938

[16] Hultin M, Gustafsson A, Hallstrom H, Johansson LA, Ekfeldt A, Klinge B (2002) Microbiological findings and host response in patients with peri-implantitis. Clin Oral Implants Res 13: 349–358. 12175371 10.1034/j.1600-0501.2002.130402.x

[17] Shibli JA, Melo L, Ferrari DS, Figueiredo LC, Faveri M, Feres M (2008) Composition of supra- and subgingival biofilm of subjects with healthy and diseased implants. Clin Oral Implants Res 19: 975–982. 10.1111/j.1600-0501.2008.01566.x 18828812

[18] Furst MM, Salvi GE, Lang NP, Persson GR (2007) Bacterial colonization immediately after installation on oral titanium implants. Clin Oral Implants Res 18: 501–508. 10.1111/j.1600-0501.2007.01381.x 17501978

[19] Quirynen M, Vogels R, Peeters W, van Steenberghe D, Naert I, Haffajee A (2006) Dynamics of initial subgingival colonization of 'pristine' peri-implant pockets. Clin Oral Implants Res 17: 25–37. 10.1111/j.1600-0501.2005.01194.x 16441782

[20] Eick S, Ramseier CA, Rothenberger K, Bragger U, Buser D, Salvi GE (2016) Microbiota at teeth and implants in partially edentulous patients. A 10-year retrospective study. Clin Oral Implants Res 27: 218–225. 10.1111/clr.12588 25827437

[21] Hagi TT, Klemensberger S, Bereiter R, Nietzsche S, Cosgarea R, Flury S, et al (2015) A Biofilm Pocket Model to Evaluate Different Non-Surgical Periodontal Treatment Modalities in Terms of Biofilm Removal and Reformation, Surface Alterations and Attachment of Periodontal Ligament Fibroblasts. PLoS One 10: e0131056 10.1371/journal.pone.0131056 26121365 PMC4486723

[22] Yamamoto A, Tanabe T (2013) Treatment of peri-implantitis around TiUnite-surface implants using Er:YAG laser microexplosions. Int J Periodontics Restorative Dent 33: 21–30. 23342343 10.11607/prd.1593

[23] Eick S, Straube A, Guentsch A, Pfister W, Jentsch H (2011) Comparison of real-time polymerase chain reaction and DNA-strip technology in microbiological evaluation of periodontitis treatment. Diagn Microbiol Infect Dis 69: 12–20. 10.1016/j.diagmicrobio.2010.08.017 21146709

[24] Eick S, Strugar T, Miron RJ, Sculean A (2014) In vitro-activity of oily calcium hydroxide suspension on microorganisms as well as on human alveolar osteoblasts and periodontal ligament fibroblasts. BMC Oral Health 14: 9 10.1186/1472-6831-14-9 24475753 PMC3915246

[25] Ando Y, Aoki A, Watanabe H, Ishikawa I (1996) Bactericidal effect of erbium YAG laser on periodontopathic bacteria. Lasers Surg Med 19: 190–200. 10.1002/(SICI)1096-9101(1996)19:2<190::AID-LSM11>3.0.CO;2-B 8887923

[26] Noiri Y, Katsumoto T, Azakami H, Ebisu S (2008) Effects of Er:YAG laser irradiation on biofilm-forming bacteria associated with endodontic pathogens in vitro. J Endod 34: 826–829. 10.1016/j.joen.2008.04.010 18570988

[27] Schwarz F, Sculean A, Romanos G, Herten M, Horn N, Scherbaum W, et al (2005) Influence of different treatment approaches on the removal of early plaque biofilms and the viability of SAOS2 osteoblasts grown on titanium implants. Clin Oral Investig 9: 111–117. 10.1007/s00784-005-0305-8 15841403

[28] Sennhenn-Kirchner S, Schwarz P, Schliephake H, Konietschke F, Brunner E, Borg-von Zepelin M (2009) Decontamination efficacy of erbium:yttrium-aluminium-garnet and diode laser light on oral Candida albicans isolates of a 5-day in vitro biofilm model. Lasers Med Sci 24: 313–320. 10.1007/s10103-008-0561-3 18458992 PMC5486503

[29] Correa MG, Oliveira DH, Saraceni CH, Ribeiro FV, Pimentel SP, Cirano FR, et al (2015) Short-term microbiological effects of photodynamic therapy in non-surgical periodontal treatment of residual pockets: A split-mouth RCT. Lasers Surg Med. 10.1002/lsm.22449 26660720

[30] Giannelli M, Formigli L, Lorenzini L, Bani D (2015) Efficacy of Combined Photoablative-Photodynamic Diode Laser Therapy Adjunctive to Scaling and Root Planing in Periodontitis: Randomized Split-Mouth Trial with 4-Year Follow-Up. Photomed Laser Surg 33: 473–480. 10.1089/pho.2015.3955 26237453

[31] Bassetti M, Schar D, Wicki B, Eick S, Ramseier CA, Arweiler NB, et al (2014) Anti-infective therapy of peri-implantitis with adjunctive local drug delivery or photodynamic therapy: 12-month outcomes of a randomized controlled clinical trial. Clin Oral Implants Res 25: 279–287. 10.1111/clr.12155 23560645

[32] Kreisler M, Kohnen W, Christoffers AB, Gotz H, Jansen B, Duschner H, et al (2005) In vitro evaluation of the biocompatibility of contaminated implant surfaces treated with an Er: YAG laser and an air powder system. Clin Oral Implants Res 16: 36–43. 10.1111/j.1600-0501.2004.01056.x 15642029

[33] Friedmann A, Antic L, Bernimoulin JP, Purucker P (2006) In vitro attachment of osteoblasts on contaminated rough titanium surfaces treated by Er:YAG laser. J Biomed Mater Res A 79: 53–60. 10.1002/jbm.a.30699 16758451

[34] Coluzzi DJ (2004) Fundamentals of dental lasers: science and instruments. Dent Clin North Am 48: 751–770, v 10.1016/j.cden.2004.05.003 15464551

[35] Leja C, Geminiani A, Caton J, Romanos GE (2013) Thermodynamic effects of laser irradiation of implants placed in bone: an in vitro study. Lasers Med Sci 28: 1435–1440. 10.1007/s10103-012-1215-z 23053251

[36] Hubbezoglu I, Unal M, Zan R, Hurmuzlu F (2013) Temperature rises during application of Er:YAG laser under different primary dentin thicknesses. Photomed Laser Surg 31: 201–205. 10.1089/pho.2012.3411 23480272

[37] Shin SI, Min HK, Park BH, Kwon YH, Park JB, Herr Y, et al (2011) The effect of Er:YAG laser irradiation on the scanning electron microscopic structure and surface roughness of various implant surfaces: an in vitro study. Lasers Med Sci 26: 767–776. 10.1007/s10103-010-0819-4 20694493

[38] Ayobian-Markazi N, Karimi M, Safar-Hajhosseini A (2015) Effects of Er: YAG laser irradiation on wettability, surface roughness, and biocompatibility of SLA titanium surfaces: an in vitro study. Lasers Med Sci 30: 561–566. 10.1007/s10103-013-1361-y 23760881

[39] Brulat N, Fornaini C, Rocca JP, Darque-Ceretti E (2013) Role of surface tension and roughness on the wettability of Er:YAG laser irradiated dentin: In vitro study. Laser Ther 22: 187–194. 10.3136/islsm.22.187 24204092 PMC3813996

[40] Krohn-Dale I, Boe OE, Enersen M, Leknes KN (2012) Er:YAG laser in the treatment of periodontal sites with recurring chronic inflammation: a 12-month randomized, controlled clinical trial. J Clin Periodontol 39: 745–752. 10.1111/j.1600-051X.2012.01912.x 22694321

[41] Sanz-Sanchez I, Ortiz-Vigon A, Herrera D, Sanz M (2015) Microbiological effects and recolonization patterns after adjunctive subgingival debridement with Er:YAG laser. Clin Oral Investig. 10.1007/s00784-015-1617-y 26419675

[42] Lopes BM, Theodoro LH, Melo RF, Thompson GM, Marcantonio RA (2010) Clinical and microbiologic follow-up evaluations after non-surgical periodontal treatment with erbium:YAG laser and scaling and root planing. J Periodontol 81: 682–691. 10.1902/jop.2010.090300 20429647

[43] Persson GR, Roos-Jansaker AM, Lindahl C, Renvert S (2011) Microbiologic results after non-surgical erbium-doped:yttrium, aluminum, and garnet laser or air-abrasive treatment of peri-implantitis: a randomized clinical trial. J Periodontol 82: 1267–1278. 10.1902/jop.2011.100660 21417591

[44] Schwarz F, Sculean A, Rothamel D, Schwenzer K, Georg T, Becker J (2005) Clinical evaluation of an Er:YAG laser for nonsurgical treatment of peri-implantitis: a pilot study. Clin Oral Implants Res 16: 44–52. 10.1111/j.1600-0501.2004.01051.x 15642030

[45] Schwarz F, Sahm N, Iglhaut G, Becker J (2011) Impact of the method of surface debridement and decontamination on the clinical outcome following combined surgical therapy of peri-implantitis: a randomized controlled clinical study. J Clin Periodontol 38: 276–284. 10.1111/j.1600-051X.2010.01690.x 21219392

