# Supplementary material for: In Vitro-Activity of Er:YAG Laser in Comparison with other Treatment Modalities on Biofilm Ablation from Implant and Tooth Surfaces
Source: PLoS One. 2017 Jan 26;12(1):e0171086. doi: 10.1371/journal.pone.0171086 (PMC5268770; doi:10.1371/journal.pone.0171086)
Supplement: S1 File — The means and SD for Figs 1–5 incl. exact p-values are presented. (DOCX) [file pone.0171086.s001.docx]

**S1 Supporting data to the Figs. 1 – 5.** The means and SD for Figs. 1-5 incl. exact p-values for ANOVA and post-hoc comparisons (only if p<0.05) are presented.

**Data Fig. 1 Killing of planktonic bacteria**

Killing of selected bacterial species and a 12-species mixture after 3*20 s of laser irradiation with a power of 70 mJ (log10 cfu)6

|  | control  mean SD | | after 3*20 s of laser irradiation with a power of 70 mJ  mean SD | |
| --- | --- | --- | --- | --- |
| *P. micra* | 5.14 | 0.04 | 5.16 | 0.02 |
| *A. actinom.* | 5.08 | 0.05 | 5.12 | 0.02 |
| *F. nucl.* | 5.02 | 0.01 | 4.99 | 0.06 |
| *P. interm.* | 5.05 | 0.04 | 5.00 | 0.06 |
| *P. gingivalis* | 5.11 | 0.01 | 5.09 | 0.02 |
| *T. forsythia* | 5.04 | 0.08 | 4.86 | 0.19 |
| mixture | 4.91 | 0.06 | 4.87 | 0.06 |

**Data Fig. 2A-B. Biofilm removal from dentine disks**

Remains of biofilm after exposing to Gracey curette (CUR), photodynamic therapy (PDT), CUR combined with PDT (CUR/PDT) and Er:YAG laser irradiation (Er:YAG)

Presented are total counts (log10 cfu; A) and counts for selected species (log10; B)

A)

|  | mean | SD | p with  control CUR PDT CUR/PDT | | | |
| --- | --- | --- | --- | --- | --- | --- |
| con | 6.92 | 0.75 |  |  |  |  |
| CUR | 5.21 | 0.64 | 0.001 |  |  |  |
| PDT | 5.16 | 0.82 | 0.002 |  |  |  |
| CUR/PDT | 2.92 | 1.90 | <0.001 | <0.001 | <0.001 |  |
| Er:YAG | 4.48 | 1.55 | <0.001 |  |  | 0.005 |

ANOVA: p<0.001

B)

|  | mean  *P. gingivalis* | SD | p with  control CUR PDT CUR/PDT | | | |
| --- | --- | --- | --- | --- | --- | --- |
| con | 4.77 | 0.81 |  |  |  |  |
| CUR | 2.23 | 1.05 | <0.001 |  |  |  |
| PDT | 2.91 | 1.41 | <0.001 |  |  |  |
| CUR/PDT | 1.71 | 1.15 | <0.001 |  | 0.008 |  |
| Er:YAG | 2.18 | 1.1 | <0.001 |  |  |  |

ANOVA: p<0.001

|  | mean  *T. forsythia* | SD | p with  control CUR PDT CUR/PDT | | | |
| --- | --- | --- | --- | --- | --- | --- |
| con | 4.67 | 1.26 |  |  |  |  |
| CUR | 1.72 | 1.90 | <0.001 |  |  |  |
| PDT | 2.72 | 1.67 | 0.003 |  |  |  |
| CUR/PDT | 1.25 | 1.56 | <0.001 |  | 0.038 |  |
| Er:YAG | 2.22 | 1.94 | <0.001 |  |  |  |

ANOVA: p<0.001

|  | mean  *P. micra* | SD | p with  control CUR PDT CUR/PDT | | | |
| --- | --- | --- | --- | --- | --- | --- |
| con | 4.50 | 0.48 |  |  |  |  |
| CUR | 3.19 | 1.33 | 0.001 |  |  |  |
| PDT | 3.58 | 1.37 | 0.026 |  |  |  |
| CUR/PDT | 3.34 | 0.80 | 0.005 |  |  |  |
| Er:YAG | 3.29 | 1.11 | 0.001 |  |  |  |

ANOVA: p=0.004

|  | mean  *F. nucleatum* | SD | p with  control CUR PDT CUR/PDT | | | |
| --- | --- | --- | --- | --- | --- | --- |
| con | 6.32 | 0.50 |  |  |  |  |
| CUR | 4.04 | 1.30 | <0.001 |  |  |  |
| PDT | 4.80 | 0.65 | 0.011 |  |  |  |
| CUR/PDT | 2.83 | 1.80 | <0.001 |  | 0.002 |  |
| Er:YAG | 3.94 | 1.34 | <0.001 |  |  | 0.049 |

ANOVA: p<0.001

**Data Fig. 3A-B. Adhesion of cells after biofilm removal from dentine disks**

Adhesion of gingival epithelial cells (A) and PDL fibroblasts (B) after biofilm removal by applying Gracey curette (CUR), photodynamic therapy (PDT), CUR combined with PDT (CUR/PDT) and Er:YAG laser irradiation (Er:YAG)

A)

|  | mean | SD | p with  con cells control CUR PDT CUR/PDT | | | | |
| --- | --- | --- | --- | --- | --- | --- | --- |
| con cells | 273 | 154 |  |  |  |  |  |
| con | 5.60 | 9.84 | <0.001 |  |  |  |  |
| CUR | 129 | 140 |  | 0.029 |  |  |  |
| PDT | 102 | 113 |  | 0.011 |  |  |  |
| CUR/PDT | 191 | 190 | 0.006 |  |  |  |  |
| Er:YAG | 101 | 76 |  | 0.010 |  |  |  |

ANOVA: p=0.004

B)

|  | mean | SD | p with  con cells control CUR PDT CUR/PDT | | | | |
| --- | --- | --- | --- | --- | --- | --- | --- |
| con cells | 107 | 42 |  |  |  |  |  |
| con | 2.60 | 3.41 | <0.001 |  |  |  |  |
| CUR | 14.2 | 21.1 |  | <0.001 |  |  |  |
| PDT | 7.98 | 10.0 |  | <0.001 |  |  |  |
| CUR/PDT | 15.4 | 10.2 |  | <0.001 |  |  |  |
| Er:YAG | 7.91 | 10.5 |  | <0.001 |  |  |  |

ANOVA: p<0.001

Data Fig. **4A-B. Biofilm removal from titanium disks**

Remains of biofilm after applying titanium curette (CUR), photodynamic therapy (PDT), titanium curette combined with PDT (CUR/PDT) and Er:YAG laser irradiation (Er:YAG)

Presented are total counts (log10 cfu; A) and counts for selected species (log10; B)

A)

|  | mean | SD | p with  control CUR PDT CUR/PDT | | | |
| --- | --- | --- | --- | --- | --- | --- |
| con | 6.64 | 0.55 | <0.001 |  |  |  |
| CUR | 4.17 | 1.21 | <0.001 |  |  |  |
| PDT | 4.08 | 0.97 | <0.001 |  |  |  |
| CUR/PDT | 2.79 | 1.27 | <0.001 | 0.010 | 0.015 |  |
| Er:YAG | 2.19 | 1.92 | <0.001 | <0.001 | 0.001 |  |

ANOVA: p<0.001

B)

|  | mean  *P. gingivalis* | SD | p with  control CUR PDT CUR/PDT | | | |
| --- | --- | --- | --- | --- | --- | --- |
| con | 5.02 | 0.93 |  |  |  |  |
| CUR | 3.32 | 1.40 | 0.005 |  |  |  |
| PDT | 3.63 | 1.25 | 0.020 |  |  |  |
| CUR/PDT | 2.19 | 1.65 | <0.001 |  | 0.018 |  |
| Er:YAG | 1.68 | 1.77 | <0.001 | 0.007 | 0.002 |  |

ANOVA: p<0.001

|  | mean  *T. forsythia* | SD | p with  control CUR PDT CUR/PDT | | | |
| --- | --- | --- | --- | --- | --- | --- |
| con | 4.81 | 0.83 |  |  |  |  |
| CUR | 1.44 | 2.15 | <0.001 |  |  |  |
| PDT | 2.48 | 2.24 | 0.001 |  |  |  |
| CUR/PDT | 0.38 | 1.28 | <0.001 |  | 0.002 |  |
| Er:YAG | 0 | 0 | <0.001 | 0.027 | <0.001 |  |

ANOVA: p<0.001

|  | mean  *P. micra* | SD | p with  control CUR PDT CUR/PDT | | | |
| --- | --- | --- | --- | --- | --- | --- |
| con | 5.52 | 0.98 |  |  |  |  |
| CUR | 3.02 | 2.39 | 0.004 |  |  |  |
| PDT | 3.82 | 2.24 | 0.044 |  |  |  |
| CUR/PDT | 3.16 | 2.19 | 0.006 |  |  |  |
| Er:YAG | 2.57 | 1.98 | 0.001 |  |  |  |

ANOVA: p=0.007

|  | mean  *F. nucleatum* | SD | p with  control CUR PDT CUR/PDT | | | |
| --- | --- | --- | --- | --- | --- | --- |
| con | 6.01 | 0.73 |  |  |  |  |
| CUR | 4.31 | 1.21 | 0.019 |  |  |  |
| PDT | 3.07 | 2.30 | <0.001 |  |  |  |
| CUR/PDT | 1.59 | 1.76 | <0.001 | <0.001 | 0.040 |  |
| Er:YAG | 2.27 | 2.09 | <0.001 | 0.005 |  |  |

ANOVA: p<0.001

**Fig. 5A-C. Adhesion of cells after biofilm removal from titanium disks**

Adhesion of gingival epithelial cells (A), gingival fibroblasts (B) and osteoblast-like cells (C) before and after biofilm removal by after applying titanium curette (CUR), photodynamic therapy (PDT), titanium curette combined with PDT (CUR/PDT) and Er:YAG laser irradiation (Er:YAG)

A)

|  | mean | SD | p with  con cells control CUR PDT CUR/PDT | | | | |
| --- | --- | --- | --- | --- | --- | --- | --- |
| con cells | 1607 | 305 |  |  |  |  |  |
| con | 107 | 127 | <0.001 |  |  |  |  |
| CUR | 649 | 320 | <0.001 | p<0.001 |  |  |  |
| PDT | 614 | 312 | 0.001 | p<0.001 |  |  |  |
| CUR/PDT | 449 | 250 | 0.016 | p<0.001 |  |  |  |
| Er:YAG | 500 | 195 | 0.006 | p<0.001 |  |  |  |

ANOVA:

B)

|  | mean | SD | p with  con cells control CUR PDT CUR/PDT | | | | |
| --- | --- | --- | --- | --- | --- | --- | --- |
| con cells | 374 | 204 |  |  |  |  |  |
| con | 163 | 176 | 0.048 |  |  |  |  |
| CUR | 211 | 150 |  |  |  |  |  |
| PDT | 116 | 122 |  | 0.016 |  |  |  |
| CUR/PDT | 284 | 148 |  |  |  |  |  |
| Er:YAG | 405 | 353 | 0.024 |  |  | 0.007 |  |

ANOVA: p=0.049

C)

|  | mean | SD | p with  con cells control CUR PDT CUR/PDT | | | | |
| --- | --- | --- | --- | --- | --- | --- | --- |
| con cells | 583 | 496 |  |  |  |  |  |
| con | 449 | 404 |  |  |  |  |  |
| CUR | 981 | 1020 |  |  |  |  |  |
| PDT | 649 | 561 |  |  |  |  |  |
| CUR/PDT | 632 | 344 |  |  |  |  |  |
| Er:YAG | 2106 | 772 | <0.001 | <0.001 | 0.002 | <0.001 | <0.001 |

ANOVA: p<0.001
